# Supplementary figures and images for: Genetic variation of the HIV-1 subtype C transmitted/founder viruses long terminal repeat elements and the impact on transcription activation potential and clinical disease outcomes
Source: PLoS Pathog. 2023 Jun 12;19(6):e1011194. doi: 10.1371/journal.ppat.1011194 (PMC10289673; doi:10.1371/journal.ppat.1011194)

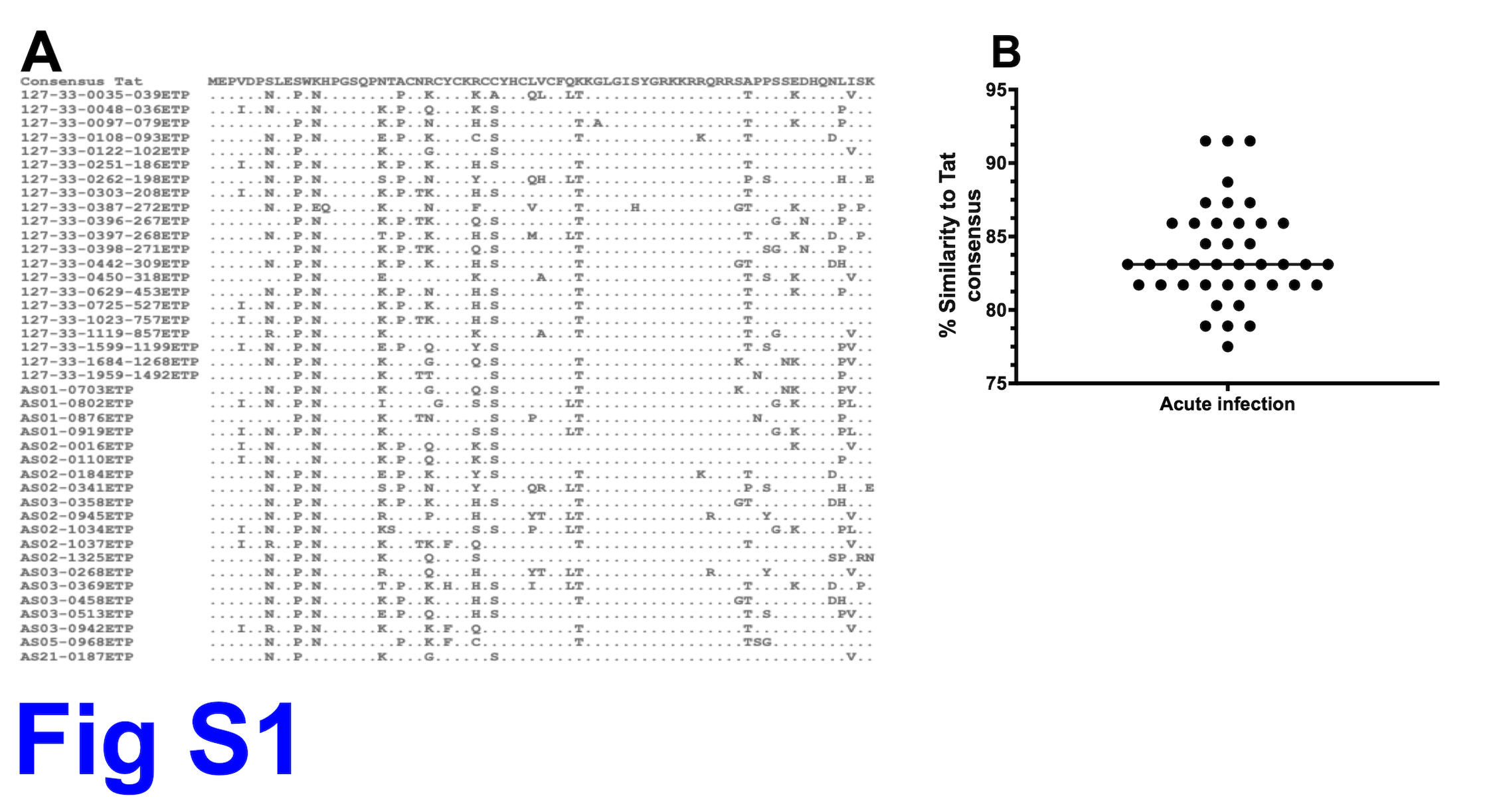

Supplement: S1 Fig — A: The patient derived Tat sequences were aligned against the Zambian subtype C consensus Tat sequence, amplified from the following reagent that was obtained through the NIH HIV Reagent Program, Division of AIDS, NIAID, NIH: Plasmid pcDNA3.1(+) Expressing Isogenic Mutant Human Immunodeficiency Virus Type 1 Subtype C BL43/02 Tat (pC-Tat.BL43.CC), ARP-11785, contributed by Dr. Udaykumar Ranga. B: Shows the percentage of similarity of each individual patients derived LTR at early time point (ETP) to the consensus sequence. (TIFF) [file ppat.1011194.s001.tiff]

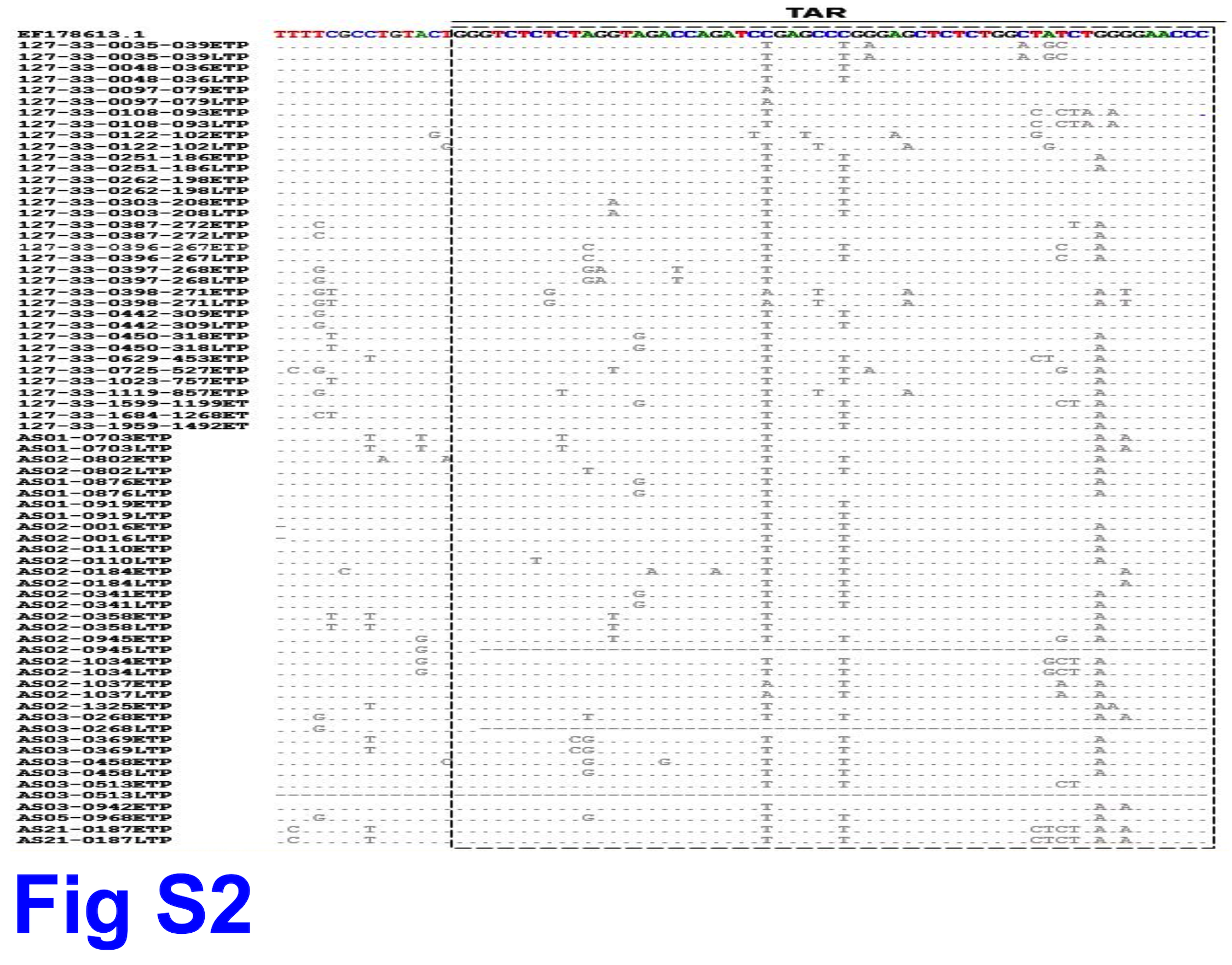

Supplement: S2 Fig — The patient derived TAR element sequences were aligned against the Indian subtype C reference sequence, EF178613.1. The dots represent nucleotide bases that are identical to the reference sequence. The doted block highlight the TAR element, TAR loop region. Overall, the T/F virus canonical sequence of the TAR loop region was relatively conserved. (TIFF) [file ppat.1011194.s002.tiff]

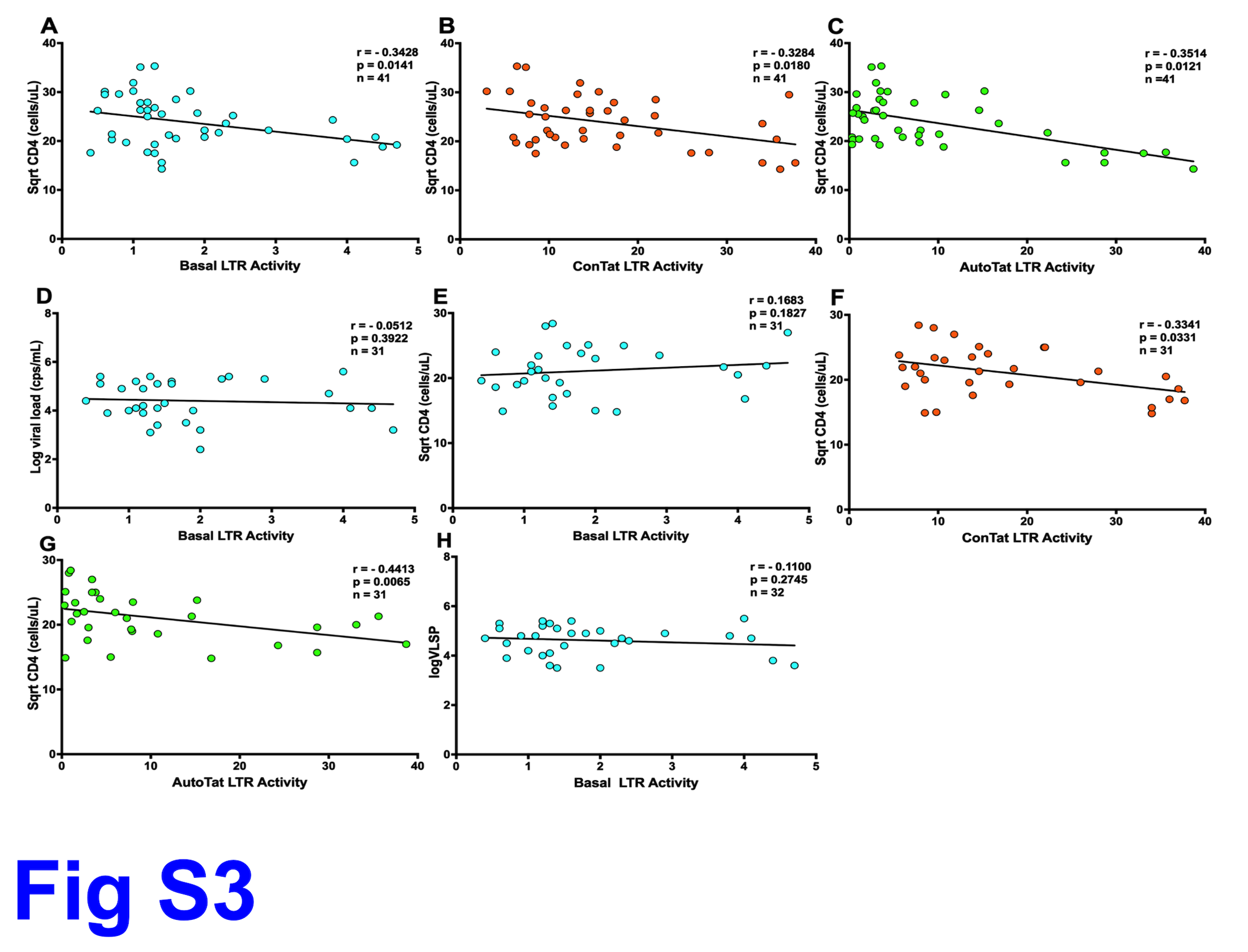

Supplement: S3 Fig — A: Basal T/F LTR transcriptional activity exhibit significant negative correlation with CD4 T cell counts at or near transmission (shown in Cyan). B: Consensus Tat-mediated T/F LTR transcriptional activity exhibit significant negative correlation with CD4 T cell counts at or near transmission (shown in orange). C: Autologous Tat-mediated T/F LTR transcriptional activity exhibit significant negative correlation with CD4 T cell counts at or near transmission (shown in green). D: Basal T/F LTR transcriptional activity shows no relationship with viral loads at one-year post infection timepoint (shown in Cyan). E: Basal T/F LTR transcriptional activity shows no relationship with CD4 T cell counts at one-year post infection (shown in Cyan). F: Consensus Tat-mediated T/F LTR transcriptional activity exhibit significant negative correlation with CD4 T cell counts at one post infection timepoint (shown in orange). G: Autologous Tat-mediated T/F LTR transcriptional activity exhibit significant negative correlation with CD4 T cell counts at one-year timepoint (shown in green). H: Basal T/F LTR transcriptional activity shows no relationship with viral load set point (shown in Cyan). * One of the ten patients without the matching plasma sample at approximately one-year post infection, had longitudinal plasma viral load measurements thus making a total of 32 patients whose viral load set point could be calculated. (TIFF) [file ppat.1011194.s003.tiff]

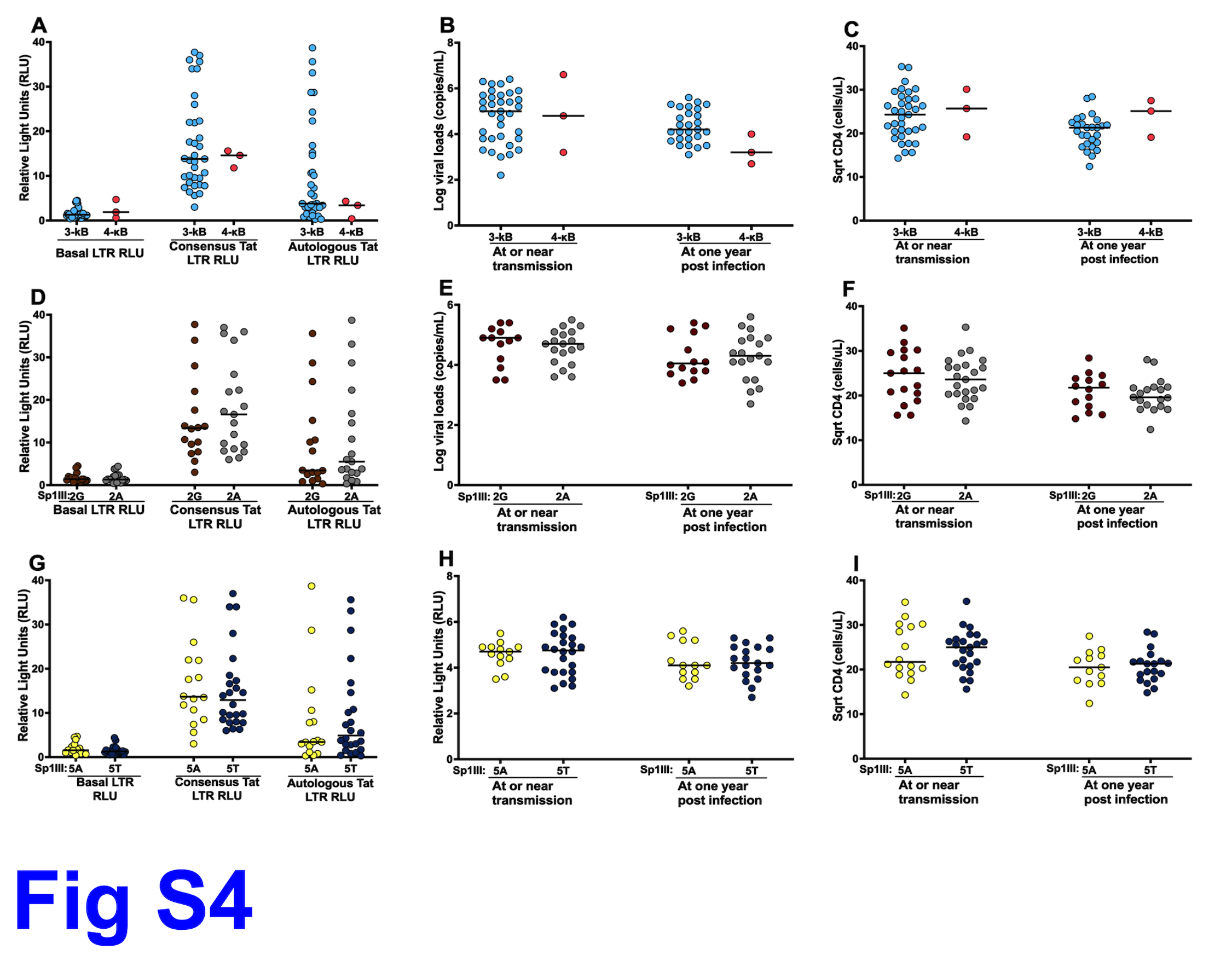

Supplement: S4 Fig — Each point in the plot represents an HIV-1 positive individual. A-C: No difference in transcriptional activity between the T/F LTR sequences exhibiting 3 NF-kB (shown in blue) versus 4 NF-kB (shown in tangerine) similarly viral loads and CD4 T cell counts were not different between patients infected with T/F viruses exhibiting 3 NF-kB sites versus 4 NF-kB sites. D-F: Transcriptional activity between the T/F LTR sequences exhibiting Sp1III_2G (shown in mocha) versus Sp1III_2A (shown in grey) were not different and similarly viral loads and CD4 T cell counts were not different between patients infected with T/F viruses Sp1III_2G versus Sp1III_2A. G-I: Transcriptional activity between the T/F LTR sequences exhibiting Sp1III_5A (shown in yellow) versus Sp1III_5T (shown in navy blue) were not different and similarly viral loads and CD4 T cell counts were not different between patients infected with T/F viruses Sp1III_5A versus Sp1III_5T. (TIFF) [file ppat.1011194.s004.tiff]
