# Supplementary material for: Genetic variation of the HIV-1 subtype C transmitted/founder viruses long terminal repeat elements and the impact on transcription activation potential and clinical disease outcomes
Source: PLoS Pathog. 2023 Jun 12;19(6):e1011194. doi: 10.1371/journal.ppat.1011194 (PMC10289673; doi:10.1371/journal.ppat.1011194)
Supplement: S1 Table — (DOCX) [file ppat.1011194.s005.docx]

**Table S1.**

| Characteristics | At viral RNA detection or enrolment | | |  | | At one-year post infection | | | | | |
| --- | --- | --- | --- | --- | --- | --- | --- | --- | --- | --- | --- |
|  | **FRESH** | **HPP Cohort** | **p-value** | |  | | **FRESH** | **HPP cohort** | **p-value** | |  |
| Number of patients (%)  Age (yrs) mean ± SD (range)  Gender, female (%)  Fiebig Stage (number of patients)  Matched samples obtained at early timepoint/late timepoint (%)  Median days post virus detection or enrolment (IQR)  CD4 T cell counts (IQR)  Median Log_10_ HIV RNA (IQR)  Median rate of CD4 T cell decline per month (IQR)  Median Log_10_ viral load set point (IQR) | 21 (51.22)  21.20 ± 1.80  21/21 (100.00)  I (15)  14/21 (66.67)  1 (IQR, 0.25–3.00)  745,54 (IQR, 447.69–910.53)  4.80 (IQR, 3.80-5.45)  −15.90 (IQR,−27.42-−2.67)  4.80 (IQR, 3.90-5.05) | 20 (48.78)  32.80 ± 11.40  13/20 (65.00)  V/VI (20)  17/20 (85.00)  34 (IQR, 30.00–40.75)  453.70 (IQR, 382.39–616.37)  5.10 (IQR, 3.90-5.78)  −6.79 (IQR,−12.01 -−0.20)  4.70 (IQR, 410-510) | 0.8714  0.2016  0.4838  N/A  0.6106  <0.0001  0.0240  0.9448  0.5855  0.9448 | |  | | 14 (45.16)  N/A  14/14 (100.00)  I (14)  N/A  N/A  490.00 (IQR, 338.50-584.50)  3.83 (IQR, 3.37-5.04)  N/A  N/A | 17 (54.84)  N/A  13/17 (76.47)  V/VI (17)  N/A  N/A  401.00 (IQR, 288.00-478.00)  3.83 (IQR, 4.05-4.88)  N/A  N/A | | 1.0000  N/A  0.7926  N/A  N/A  N/A  0.6259  0.8339  N/A  N/A |  |

- N/A means the p-value could not be calculated or the information at viral RNA detection or enrolment timepoint is the same as the information at one-year post information.
- IQR (interquartile range).
