# Supplementary material for: Genetic variation of the HIV-1 subtype C transmitted/founder viruses long terminal repeat elements and the impact on transcription activation potential and clinical disease outcomes
Source: PLoS Pathog. 2023 Jun 12;19(6):e1011194. doi: 10.1371/journal.ppat.1011194 (PMC10289673; doi:10.1371/journal.ppat.1011194)
Supplement: Attachment — Submitted filename: Rebuttal-Genetic variation_HIV LTR02May23.pdf [file ppat.1011194.s006.pdf]

HIV Pathogenesis Programme
Doris Duke Medical Research Institute
Nelson R Mandela School of Medicine

UNIVERSITY OF KWAZULU-NATAL
INYUVESI YAKWAZULU-NATALI

Private Bag 7, Congella, 4013
DDMRI, Level 2, Room 209/210
719 Umbilo Road, DURBAN
4001 South Africa

02 May 2023

Title: Genetic variation of the HIV-1 subtype C transmitted/founder viruses long terminal repeat elements and the impact on transcription activation potential and clinical disease outcomes

Corresponding Author: Paradise Madlala

Dear Editor,

Hereby I resubmit the revised version of the manuscript entitled “Genetic variation of the HIV-1 subtype C transmitted/founder viruses long terminal repeat elements and the impact on transcription activation potential and clinical disease outcomes”.

We would like to acknowledge anonymous reviewers #1 and #2 for their comments and suggestions, which have helped to improve the manuscript. We considered the comments with care and acted on the points raised by the reviewers on the following pages:

- page 5, paragraph 1, lines 82, 89 – 91,
- page 6, paragraph 1, line 97,
- page 8, paragraph 1, lines 140, 147; 150 – 156,
- page 10, below Table 1, line 169,
- page 11, paragraph 2, lines 182 – 189,

- page 12, paragraph 1, line 204,
- page 15 paragraphs 2&3, lines 269, 270, 274, 276 & 282,
- page 16 paragraph 1, lines 288 -299,
- page 19, paragraph 2, line 370,
- page 20, paragraph 3, lines 392 – 394,
- page 25, paragraph 3, lines: 500 – 503
- page 26, paragraph 1, lines: 507- 522,
- page 27, paragraph 1, lines 532 – 537,
- page 30 paragraph 1, lines 596 – 597,
- page 37 Figure 5 legend, lines 449 – 352,
  Figure S1 legend, lines 354 – 362,
  Figure S2 legend, lines 364 - 268
  Figure S3 legend, line 370
  Figure S4 legend, line 387
- page 38 Table title S1, lines 903 - 904

Below, please find our answers to all reviewer's comments, presented in the same order as in the report.

Yours Sincerely,

Paradise Madlala

Faculty and Senior Lecturer at HIV Pathogenesis Programme (HPP) of the University of KwaZulu-Natal

Reviewer #1

Part I - Summary:

This a longitudinal analysis of HIV subtype C LTR diversity and transcriptional activation during acute/early infection in South Africa. The patient population, in vitro methods, and data analysis are well described. Overall, this is a very nice study with interesting findings.

Response:

- **We are grateful to the reviewer for their insightful comments on our paper.**

Part II – Major Issues: Key Experiments Required for Acceptance:

N/A.

Response:

- **We are grateful to the reviewer for their insightful comments on our paper.**

Part III – Minor Issues: Editorial and Data Presentation Modifications:

A few minor revisions would strengthen this manuscript further, including:

- *Lines 145 and 148 and Table 1: Why is the median square root CD4 T cell count presented rather than the absolute value?*

Response:

An analysis showing the absolute CD4 T cell Count has been included in this article as supporting Table S1 and is referenced on page 8 lines 147 and 151. Furthermore, the following sentence has been added in line 152 – 156, "While the median absolute CD4 T cell count was significantly different between the group whose samples were available within a median of 1 day of virus detection and 34 days post infection at the earliest time point (p=0.0240), the median square root CD4 T cell count is reported in this study because a square root transformation has the effect of making the data less skewed and variation more uniform." The title of the supporting table is on page 39 lines 874 – 875, "Table S1: The demographic and clinical characteristics showing absolute CD4 T cell count of the 41 people living with HIV-1 used in this study."

- **.**

- *Line 178: what is the range of pairwise diversity between the early and late time points? Were there any paired samples that showed no / very little inter-time point diversity?*

Response:

- **On page 11 in paragraph 2, we have now revised lines 185 - 189 to reflect the range of inter-patient T/F LTR sequence diversity and added a new sentence to reflect the range of pairwise diversity between early and late time points. Therefore, the sentences read as follows now, "Furthermore, our data demonstrate that there was 5.7% (Range: 2 – 12%) inter-patient T/F LTR sequence diversity with subsequent intrahost viral evolution observed in 48.4% of the participants analyzed at 12 months post-infection. The pairwise diversity between the early (acute) and late timepoints was 0.03% (Range: -2 – 5) with 51.6% of the sequences exhibiting no inter-timepoint diversity."**

- *Additional details on consensus versus autologous Tat sequences are needed. Were autologous Tat sequences derived from a previous study? How similar / different are they from the consensus Tat sequence?*

Response:

- **More details about HIV-1 subtype C consensus LTR sequence used for transcription assay has been added on page 24 line 491 - 495. Now the sentence reads as follows "The HIV-1C consensus LTR sequence obtained from NIH AIDS Reagent Program (catalogue number: 4789) or remainder of gel extracted DNA amplified from LTR U3R of the 41 RNA samples obtained at the earliest post infection timepoint (referred to as T/F LTR) were cloned into the pGL3 Basic vector expressing Firefly Luciferase (Promega Corporation, Wisconsin, USA) as previously described (63)".**

- **More details about HIV-1 subtype C consensus Tat sequence used for the transcription assay have been added on page 25 lines 518 - 524. Now the sentence reads as follows ". A plasmid DNA pool of 400 ng, containing 300 ng of the recombinant T/F LTR-pGL3 plasmid and 100 ng of**

recombinant control insert DNA-pTarget plasmid (Promega Corporation, Madison, WI) for the Tat minus transfection or 100 ng of recombinant HIV-1C consensus Tat sequence obtained from pcTat.BL43.CC (NIH AIDS Reagent Program, catalogue number: 11785) or autologous Tat-pTarget plasmid (obtained from the same sample from which the LTR was derived, Mkhize et al., *unpublished data*) was prepared in 50 µL of serum-free RPMI medium".

- *The diversity inherent in Figure 2 is difficult to see. A phylogram may be better than a circular tree for data visualization / presentation.*

Response:

- **Figure 2 has been changed to a phylogram to improve data visualization/ presentation.**

- *The use of Jurkat cells is appropriate. However, showing some results in another cell type would be helpful for confirming that results are not cell type or cell line dependent.*

Response:

- **The use of only one cell line has now been acknowledged as one of the limitations of the study. To this effect, a new sentence has been added on page 19 lines 383 – 385 "While only one cell line was used in this study, it is possible that the use of other cell lines may have yielded same results thus confirming that these results are not cell type dependent or shown different results."**

Reviewer #2

Part I - Summary:

Strengths

Detailed characterisation of the genetic evolution of the HIV LTR from subtype C strains in the first year after acute infection in 41 patients with a focus on transcriptional activity. Numbers on genetic diversity and evolution are given. A correlation between transcriptional activity and viral loads is demonstrated. Heterogeneous response to LRA activation is shown. No evidence for selection of 4 NF-kappaB sites in the LTR in South African population in contrast to previous reports from India.

Response:

- **We are grateful to the reviewer for their insightful comments on our paper.**

Part II – Major Issues: Key Experiments Required for Acceptance:

No additional experiments required.

Response:

- **We are grateful to the reviewer for their insightful comments on our paper.**

Part III – Minor Issues: Editorial and Data Presentation Modifications:

- *Please rephrase line 175-177 on page 6. Not clear what authors want to tell.*

  Response:

- **The sentence on page 10 lines 175 – 177 has been paraphrased as follows "The phylogenetic tree shows intra-patient clustering of LTR nucleotide sequences obtained at acute and one-year timepoints and that LTR sequences generated at the acute timepoint, without matching later timepoint samples, were distinct and did not form clusters".**

- *Line 360: perhaps a number is missing (4 NF-kappa B sites)*

  Response:

- **The sentence on page 18 lines 360 – 362 has been paraphrased as follows "However, our study did not show that viruses with 4 NF-kB binding sites**

are being preferentially transmitted or have superior transcriptional potential".

----

1) We ask that a manuscript source file is provided at Revision. Please upload your manuscript file as a .doc, .docx, .rtf or .tex. If you are providing a .tex file, please upload it under the item type 'LaTeX Source File' and leave your .pdf version as the item type 'Manuscript'.

Response:

- **A manuscript source file is provided and oour manuscript file has been uploaded as a .docx file.**

2) Please ensure that the CRediT author contributions listed for every co-author are completed accurately and in full. At this stage, "All Authors" require contributions. Please ensure that the full contributions of each author are acknowledged in the ""Add/Edit/Remove Authors"" section of our submission form.

Response:

- **The CRediT author contributions listed for every co-author are completed accurately and in full. The full contributions of each author are acknowledged in the ""Add/Edit/Remove Authors"" section of our submission form**

3) Please upload all main figures as separate Figure files in .tif or .eps format. For more information about how to convert and format your figure files please see our guidelines:

Response:

- **All main figures are uploaded as separate Figure files in .tif format.**

4) Please amend your detailed Financial Disclosure statement. This is published with the article, therefore should be completed in full sentences and contain the exact wording you wish to be published.
i). State what role the funders took in the study. If the funders had no role in your study, please state: "The funders had no role in study design, data collection and analysis, decision to publish, or preparation of the manuscript."
ii). If any authors received a salary from any of your funders, please state which authors and which funders.

Response:

- ***Our detailed Financial Disclosure has been amended in full sentences to contain the exact wording we wish to be published.***

5) Please ensure that the funders and grant numbers match between the Financial Disclosure field and the Funding Information tab in your submission form. Note that the funders must be provided in the same order in both places as well. Currently, " The UK Foreign, Commonwealth & Development Office"is missing from the Funding Information.

Response:

- **The funders and grant numbers match between the Financial Disclosure field and the Funding Information tab in our submission form.**

6) We noticed that you used "unpublished data" in the manuscript. We do not allow these references, as the PLOS data access policy requires that all data be either published with the manuscript or made available in a publicly accessible database. Please amend the supplementary material to include the referenced data or remove the references.

Response:

- **More details about HIV-1 subtype C consensus LTR sequence used for transcription assay has been added on page 24 line 491 - 495. Now the sentence reads as follows "The HIV-1C consensus LTR sequence obtained from NIH AIDS Reagent Program (catalogue number: 4789) or remainder of gel extracted DNA amplified from LTR U3R of the 41 RNA samples obtained at the earliest post infection timepoint (referred to as T/F LTR) were cloned into the pGL3 Basic vector expressing Firefly Luciferase (Promega Corporation, Wisconsin, USA) as previously described (63)".**

- **More details about HIV-1 subtype C consensus Tat sequence used for the transcription assay have been added on page 25 lines 518 - 524. Now the sentence reads as follows ". A plasmid DNA pool of 400 ng, containing 300 ng of the recombinant T/F LTR-pGL3 plasmid and 100 ng of recombinant control insert DNA-pTarget plasmid (Promega Corporation, Madison, WI) for the Tat minus transfection or 100 ng of recombinant HIV-**

1C consensus Tat sequence obtained from pcTat.BL43.CC (NIH AIDS Reagent Program, catalogue number: 11785) or autologous Tat-pTarget plasmid (obtained from the same sample from which the LTR was derived, Mkhize et al., *unpublished data*) was prepared in 50 µL of serum-free RPMI medium".

7) We do not publish any copyright or trademark symbols that usually accompany proprietary names, eg (R), (C), or TM (e.g. next to drug or reagent names). Please remove all instances of trademark/copyright symbols throughout the text, including SuperScript™ on line no 461,Platinum™ on line no 462, SuperScript™ ,Platinum™on line no 467.

Response:

- **All copy or trademark symbols have been removed. SuperScript™ has been changed to SuperScript on page 24 line 461, Platinum™ has been changed to Platinum on page 24 line 462, SuperScript™ has been changed to Superscript on page 24 line 470, Platinum™ has been changed to Platinum on page 24 line 470.**

8) We have noticed that you have included SI Legends but (Figure S3)legend is missing .Please add a full list of legends for your Supporting Information file after the references list.

Response:

- **On page 37 lines 874 – 889, Figure S3 legend has been added together with the full list (page 37, lines 858 – page 38, line 902) for our Supporting information file.**

9) Please make sure that the label in the supplementary legends matches the label in the uploaded Supplementary files (Table S1).

Response:

- **The supplementary legends matches the label in the uploaded Supplementary files (Table S1)**
